# Ketogenic Diet in Children with Type 1 Diabetes: Parental Motivations and Potential Risks for Metabolic Health and Development

**DOI:** 10.3390/nu18081244

**Published:** 2026-04-15

**Authors:** Rujith Kovinthapillai, Yung-Yi Lan, Andrzej Kędzia, Elżbieta Niechciał

**Affiliations:** 1 Center for Medical Education in English, Poznan University of Medical Sciences, 41 Jackowskiego St., 60-512 Poznań, Poland; rujithk22@icloud.com (R.K.); catherine974u@gmail.com (Y.-Y.L.); 2 Department of Pediatric Diabetes, Auxology and Obesity, Poznan University of Medical Sciences, ul. Szpitalna 27/33, 60-572 Poznań, Poland; akedzia@ump.edu.pl

**Keywords:** ketogenic diet, type 1 diabetes, children, parental decision-making, dyslipidemia, growth, safety

## Abstract

**Background:** The ketogenic diet has gained substantial popularity in recent years, and an increasing number of caregivers of children with type 1 diabetes are considering it as a nutritional strategy to improve glycemic control. Reported benefits include fewer postprandial glucose fluctuations, lower insulin requirements, and reduced insulin-associated weight gain. However, the use of this diet in children with type 1 diabetes remains highly debated, and scientific evidence regarding its safety and long-term effects in the pediatric population is limited. This narrative review aims to explore the motivations that lead parents to initiate a ketogenic diet in their children with type 1 diabetes and to summarize current knowledge on its potential metabolic and developmental consequences. **Methods:** A narrative review of the literature was conducted, including original research articles, case reports, and existing reviews addressing the use of ketogenic diets in children with type 1 diabetes. Clinical observations and published accounts of family experiences were also examined to contextualize emerging concerns and motivations. **Results:** Parents most commonly adopt a ketogenic diet for their children due to the desire for tighter glucose control, concerns about insulin-related weight gain, and the influence of information shared on social media. Some observational data suggest improvements in glycemic stability and reduced insulin requirements under ketogenic dietary regimens, while available evidence also highlights several potential risks, including dyslipidemia, increased susceptibility to hypoglycemia, slowed linear growth, and possible neurocognitive and psychosocial effects. Long-term safety data remain scarce, and current findings are insufficient to establish clear clinical recommendations. **Conclusions:** Interest in ketogenic diets among families of children with type 1 diabetes is growing, yet existing evidence suggests that the diet may pose significant metabolic and developmental risks in this population. Further well-designed studies are needed to evaluate its safety and efficacy. This review may assist clinicians in counseling families and underscores the need for evidence-based guidelines regarding restrictive dietary patterns in youth with type 1 diabetes.

## 1. Introduction

Ketogenic diets (KDs) have gained widespread attention and use in the general population over the past decade. Originally developed as a treatment for refractory epilepsy in the 1920s, the KD has been increasingly promoted through social media platforms and popular nutrition narratives for non-epilepsy indications, including weight loss, metabolic health, and glycemic control [1,2,3]. The core principle rests on marked carbohydrate restriction and a compensatory increase in fat intake, a combination aimed at inducing nutritional ketosis and limiting postprandial glucose excursions [3,4]. Such dietary approaches have drawn interest not only in adults with metabolic disorders but also among caregivers of children and adolescents with chronic diseases such as type 1 diabetes (T1D) [4,5].

In pediatric T1D, optimal glycemic control remains a central goal to minimize the risk of acute, life-threatening complications and long-term microvascular and macrovascular outcomes [6]. While technological advancements in insulin formulations and delivery methods, continuous glucose monitoring (CGM), and hybrid closed-loop systems have contributed to improved T1D management, dietary choices continue to be a key modifiable factor influencing day-to-day glycemic variability [6,7,8]. In this context, some caregivers have increasingly considered ketogenic or very-low-carbohydrate diets for children with T1D, motivated by reported benefits of lowered insulin requirements, reduced postprandial hyperglycemia, improved glycemic stability, and perceived improvements in quality of life [5,9]. Additionally, online discussions have further fueled interest in carbohydrate restriction as a means of achieving near-normoglycemia, despite limited long-term safety and efficacy data in pediatric populations [5,10].

Current nutritional guidance from major diabetes organizations, such as the American Diabetes Association (ADA) and the International Society for Pediatric and Adolescent Diabetes (ISPAD), recommends a balanced and flexible macronutrient intake for children and adolescents with T1D. These guidelines advocate adequate carbohydrates tailored to insulin dosing and emphasize a balanced nutrient intake, encompassing non-starchy vegetables, whole grains, fruits, fiber, and lean protein sources, with individualized medical nutrition therapy (MNT) that reflects the child’s developmental, cultural, and psychosocial needs, as well as family preferences [6,11,12]. Importantly, nutrition therapy in pediatric T1D seeks to balance optimal glycemic control with the support of normal growth, pubertal development, skeletal health, and neurocognitive maturation [4,10]. Within this framework, restrictive dietary patterns should be approached with caution.

The use of KDs in growing children remains a subject of debate. As stated by the American Academy of Pediatrics (AAP), potential adverse effects include dyslipidemia, hypoglycemia, ketoacidosis, nutrient deficiencies, linear growth impairment, and compromised bone metabolism [4,13,14]. Apart from metabolic consequences, restrictive diets may affect neurocognitive development, eating behaviors, and psychosocial well-being, especially during key developmental stages in childhood and adolescence [13,15]. Despite growing adoption, robust evidence regarding the safety, sustainability, and long-term efficacy of KDs in children with T1D remains scarce, with existing data mostly derived from small case series, observational studies, or adult extrapolations [5,10,13].

Given the widening gap between clinical recommendations and real-world dietary practices in pediatric T1D, there is a need for a thorough, critical evaluation of this topic. Unlike prior reviews focusing primarily on metabolic outcomes, this narrative review integrates parental and caregiver motivations for initiating and implementing KDs in children and adolescents with T1D, examines real-world implementation patterns, and assesses the metabolic, developmental, and safety implications of KDs. In addition, this review synthesizes available data and clinical observations to support clinicians in counseling families and engaging in shared decision-making, while addressing areas where additional research is warranted to inform evidence-based nutritional strategies for children with T1D.

## 2. Materials and Methods

This review used a narrative approach rather than a systematic literature review, selected to allow for a comprehensive and clinically oriented synthesis of heterogeneous evidence, including observational studies, clinical guidelines, systematic reviews, and real-world data, which may not be suitable or amenable for formal quantitative aggregation. This method is particularly relevant in exploring qualitative results, such as parental motivations, assessing metabolic, developmental, and safety implications of KDs, aiding clinicians in providing informed guidance, or addressing complex areas such as ketogenic dietary interventions in pediatric T1D, where study designs, populations, and outcome measures are highly variable. Each author independently identified and critically appraised the literature concerning KDs and their usage in pediatric T1D populations.

Any discrepancies regarding study selection or interpretation were resolved through discussion and consensus among the authors. It is acknowledged that narrative reviews are inherently subject to limitations, including potential selection bias and the absence of standardized quality assessment. To mitigate these limitations, priority was given to higher levels of evidence, such as systematic reviews, international guidelines, original research, and large observational studies, and sources were independently evaluated to support clinical guidance.

Literature searches were conducted between January 2026 and March 2026 across multiple databases, including PubMed, EMBASE, Scopus, Web of Science, and Google Scholar. Keywords encompassed terms such as “ketogenic diet,” “type 1 diabetes,” “management of type 1 diabetes,” “children and adolescents,” “low-carbohydrate,” “very-low-carbohydrate,” “micronutrient,” “macronutrient,” “nutritional status,” “glycemic control,” “parental experience,” “parental motivation,” “decision-making,” “diabetic complication,” “diabetic ketoacidosis,” “nutritional ketosis,” “growth,” “development,” “long-term effects,” and “efficacy and safety,” among others. The primary selection of studies was based on title and abstract screening for relevance to the topic, followed by full-text assessment. In addition, a snowballing technique was applied to identify additional relevant literature.

Studies published between 2006 and 2026 were considered. However, selected landmark studies published outside the specified time frame were included due to their relevance to this review’s objectives and to ensure comprehensive coverage and adequate historical context. The inclusion criteria encompassed clinical research, narrative and systematic reviews, and meta-analyses investigating the KD in children and adolescents with T1D. The exclusion criteria included studies with insufficient data or unavailable full texts, non-English language publications, non-peer-reviewed articles, duplicate publications, and abstract-only reports. In addition to data synthesis, attention was given to critically appraising study design, population characteristics, and potential sources of bias, including sample size, observational studies, and extrapolation from non-T1D populations. These limitations were considered in the interpretation of findings throughout the review. The selected literature provides theoretical insights and practical frameworks relevant to KDs and their application in pediatric T1D, with the goal of supporting clinical practice and education in pediatric T1D care.

## 3. Overview of the Ketogenic Diet

The KD refers to a nutritional approach in which carbohydrate intake is substantially limited, while fat intake predominates and protein intake is moderate. Within this framework, it is important to note that ketogenic diets belong to the broader family of low-carbohydrate dietary patterns. Low-carbohydrate diets (LCDs) are generally defined as eating strategies that restrict daily carbohydrate intake to below approximately 130 g. Ketogenic diets represent the most restrictive end of this spectrum, typically limiting carbohydrate consumption to about 20–50 g per day, roughly 5–10% of total daily caloric intake, while maintaining high fat intake and adequate protein [16]. Such a macronutrient profile creates the metabolic conditions necessary for the development of nutritional ketosis. This macronutrient distribution triggers nutritional ketosis, in which circulating ketone bodies, particularly β-hydroxybutyrate, acetoacetate, and acetone, become the primary fuel source instead of glucose [17]. Importantly, a clear distinction should be made between physiological nutritional ketosis and diabetic ketoacidosis (DKA). Nutritional ketosis represents a normal adaptive response to fasting or carbohydrate restriction, characterized by normal glucose concentration (typically 70–99 mg/dL), mild ketone elevation (0.5–3 mmol/L), and no acid-base derangement [18,19]. In contrast, DKA, as defined by the ISPAD guidelines, involves simultaneous occurrence of hyperglycemia (>200 mg/dL), metabolic acidosis (venous pH < 7.3 or serum bicarbonate <18 mmol/L), and ketonemia (β-hydroxybutyrate ≥ 3.0 mmol/L) [20].

In clinical practice, particularly for epilepsy and metabolic disorders, four principal types of KD therapies (KDTs) exist, differing in their degree of carbohydrate restriction, the fat-to-combined protein and carbohydrate ratio, and dietary rigidity. These include the classic KD (CKD), medium-chain triglyceride KD (MCTKD), modified Atkins diet (MAD), and low glycemic index treatment (LGIT) [21]. The CKD represents the most restrictive variant, which relies heavily on long-chain triglycerides and is formulated at a 4:1 or 3:1 ratio of fat to combined protein and carbohydrate by weight, with carbohydrates contributing 5–10% or less of total energy intake. The regimen is most commonly prescribed for children with drug-resistant epilepsy [22]. The MCTKD replaces a portion of long-chain fats with medium-chain triglycerides, about 60% octanoic acid, and 40% decanoic acid, permitting higher carbohydrate and protein intake while maintaining ketosis via enhanced ketone production [23]. The MAD offers a more flexible and pragmatic ketogenic alternative, typically structured around a 1:1 to 2:1 ratio and about 10% of energy from carbohydrates [24]. This approach allows for 10–30 g/day of carbohydrates without strict food weighing, supporting its broader clinical applicability and making it particularly suitable for outpatient care and adult populations [25]. The LGIT emphasizes carbohydrates with a glycemic index below 50 and allows for a higher carbohydrate intake (approximately 40–60 g/day). While preserving ketosis, this strategy has been shown to improve adherence and reduce adverse effects [26]. Selection of the appropriate dietary strategy is individualized, guided by patient age, disease condition, lifestyle, and expected adherence.

Within pediatric care, KDs are an established therapeutic option for refractory epilepsy and have demonstrated efficacy in reducing seizure frequency among patients who fail to respond adequately to pharmacological therapy [27]. KDTs are generally initiated and overseen by a multidisciplinary team of pediatric neurologists, dietitians, and nurses. Close monitoring of growth parameters, nutritional status, metabolic panel and electrolytes, lipid profiles, micronutrient supplementation, ketosis levels, and potential adverse effects, such as gastrointestinal (GI) symptoms, hyperlipidemia, nephrolithiasis, and growth deceleration, are necessary to modify the diet based on clinical response and tolerance [4,28,29]. Other potential clinical applications in pediatrics include certain inborn errors of metabolism (e.g., glucose transporter protein 1 (GLUT-1) deficiency and pyruvate dehydrogenase complex deficiency), certain neurologic disorders (e.g., autism spectrum disorder (ASD), tuberous sclerosis, Dravet syndrome), overweight and obesity, tumors, and T1D; however, supporting evidence outside epilepsy is relatively sparse [29,30].

Importantly, the use of ketogenic and very-low-carbohydrate diets (VLCDs) in children with T1D differs fundamentally from their established indications. In epilepsy, ketosis is the intended therapeutic mechanism, exerting anticonvulsant effects through metabolic modulation and synaptic stabilization within the central nervous system, whereas carbohydrate restriction in T1D is primarily aimed at moderating postprandial glycemic variability and insulin demand [13,27]. Moreover, KDs adopted by families and caregivers are frequently self-initiated, without formal clinical oversight or standardized frameworks, leading to substantial heterogeneity in composition, duration, and level of restriction [13,15]. Consequently, assessment of developmental, nutritional, and psychosocial outcomes may be irregular or insufficient.

These differences highlight the need to distinguish between medically supervised KDTs for defined pediatric indications from parent-initiated dietary practices in children with T1D. Recognizing this distinction is critical for interpreting current evidence, counseling families, and evaluating safety implications in T1D.

## 4. Nutritional Management of Type 1 Diabetes in Children

Nutritional management is a cornerstone of care in children with T1D, with the primary objectives being optimal glycemic control, normal growth and development, and psychosocial well-being. Unlike adults, children with T1D must balance insulin therapy not only with food intake but also with growth spurts, puberty, and evolving cognitive and emotional needs. Both the ISPAD and the ADA emphasize that nutrition therapy in pediatric diabetes should be individualized, culturally sensitive, and developmentally appropriate, integrating family education and support from trained dietitians [11,12].

Achieving tight glycemic control depends heavily on managing dietary carbohydrates, as they are the primary macronutrient that affects postprandial blood glucose. Carbohydrate counting is the preferred method endorsed by ISPAD and ADA for matching insulin dosing with food intake, especially in intensive insulin regimens [12,31]. For children on fixed insulin doses, consistent carbohydrate intake is recommended to reduce glycemic variability. Importantly, VLCDs (<50 g/day) are discouraged in pediatric populations due to potential adverse effects on growth, ketone production, and neurodevelopment [12,32].

Adequate carbohydrate intake is not only critical for metabolic stability but also for normal physical and neurological development. Carbohydrates provide the energy required for tissue building during periods of rapid growth, particularly in early childhood and adolescence. The brain relies heavily on glucose as its primary fuel, and prolonged hypoglycemia or suboptimal carbohydrate intake has been linked to impaired cognition and academic performance in youth with T1D [11,33]. Hence, nutritional strategies must ensure sufficient carbohydrate availability to support both glycemic control and developmental needs.

Psychosocial aspects are increasingly recognized as integral to effective nutritional management. Restrictive diets or inflexible eating patterns may contribute to anxiety, disordered eating, and impaired quality of life. Both ISPAD and ADA recommend flexible, inclusive approaches that encourage healthy relationships with food, promote autonomy in self-management, and involve caregivers in supportive roles [34]. Overall, nutritional care in pediatric T1D must be dynamic, developmentally appropriate, and psychosocially attuned to ensure the best possible outcomes.

## 5. Parental Motivations for Initiating a Ketogenic Diet

### 5.1. Desire for Improved Glycemic Control

A primary reason parents adopt KDs is to improve glycemic outcomes. Standard high-carbohydrate diets, even when combined with modern insulin regimens and CGM, often result in postprandial hyperglycemia and increased glycemic variability. Parents report that KDs or VLCDs markedly reduce postprandial glucose excursions, enabling tighter overall glucose control [35]. In a large self-reported study by Lennerz et al. involving 316 individuals with T1D, many of whom were children, the mean reported hemoglobin A1c (HbA1c) was 5.67%, with more than 80% of participants expressing satisfaction with blood glucose stability [35].

Clinical data provide limited but encouraging support for these observations. Ranjan et al. conducted a short-term randomized crossover study in adults with T1D and found that a low-carbohydrate diet reduced mean glucose levels and glycemic variability [36]. Although pediatric data remain sparse, parents often interpret these findings, together with their own child’s CGM trends, as sufficient justification for dietary experimentation. Families frequently describe improved time in range, reduced need for correction boluses, and fewer glucose alarms, all of which contribute to a greater sense of predictability and control [4].

Another commonly cited benefit is a reduction in total daily insulin requirements. In very low carbohydrate states, endogenous glucose production becomes the dominant glucose source, allowing for substantially lower bolus insulin doses. Some parents view this reduction as simplifying insulin management and decreasing the risk of dosing errors and hypoglycemia [37]. Lower insulin requirements are also interpreted by some caregivers as reducing metabolic strain on the body, although this concept has not been formally established in clinical research.

### 5.2. Concerns Related to Insulin Therapy

Parental skepticism regarding insulin therapy itself also contributes to the decision to pursue KDs. Insulin-associated weight gain is a well-documented phenomenon, particularly in adolescents, and can be a source of distress for both children and caregivers [38]. Families with adolescent girls in particular may express concern about the psychosocial consequences of weight gain, including body image distress, worsening insulin resistance, and social stigma. These concerns may be intensified by cultural norms that emphasize thinness and health optimization, leading families to adopt restrictive dietary approaches as a preventive strategy.

Hypoglycemia represents another central concern. Nocturnal hypoglycemia, in particular, is a major source of fear and emotional burden for caregivers. Lawton et al. conducted in-depth interviews with parents and found that fear of nighttime hypoglycemia led to chronic sleep deprivation, persistent vigilance, and compensatory behaviors involving food intake or insulin adjustment [39]. Some parents adopt the KD believing that reduced carbohydrate intake will promote more stable overnight glucose levels and lower hypoglycemia risk, even though KDs may paradoxically impair the body’s natural counter-regulatory responses to hypoglycemia.

The cognitive and logistical demands of carbohydrate counting also contribute to parental frustration. Managing meals, school lunches, social events, and insulin-to-carbohydrate ratios can be overwhelming. The KD is perceived by some parents as reducing this burden by limiting food variability and decreasing the need for frequent mealtime insulin adjustments [37]. This perceived simplification is particularly attractive to caregivers balancing diabetes management with employment, care of other children, and additional responsibilities.

### 5.3. Influence of Social Media and Online Communities

The influence of social media and online peer networks has become an increasingly important factor shaping parental decision-making. Platforms such as Reddit, Facebook, and YouTube host active communities of individuals with T1D and caregivers who advocate ketogenic and low-carbohydrate diets. These spaces are filled with anecdotal success stories, often accompanied by CGM screenshots, HbA1c results, and personal testimonials, which portray the KD as a transformative alternative to guideline-based care [40].

For many parents, these online communities offer both practical guidance, such as recipes and macronutrient strategies, and emotional reassurance. Accounts from other caregivers who report successful implementation of the KD can be validating and empowering, particularly for families who feel conventional dietary advice has failed their child. It is also noted that families may place greater trust in peer communities than in healthcare professionals, especially when clinicians express skepticism or fail to engage constructively with dietary concerns [41].

At the same time, these communities can promote mistrust of conventional medical care. Parents may perceive healthcare providers as uninformed or resistant to emerging dietary approaches, which can further distance families from multidisciplinary diabetes care teams [42]. The peer-driven culture of these groups encourages experimentation and offers social reinforcement for adherence, fostering a dietary identity that may be difficult to abandon even when medical risks are acknowledged.

While these platforms provide community support and practical guidance, they may also introduce bias through selective reporting of positive outcomes, underrepresentation of adverse effects, and amplification of anecdotal success stories. This can contribute to an overly optimistic perception of ketogenic dietary approaches and may influence decision-making in the absence of balanced or evidence-based information.

### 5.4. Psychosocial and Family Factors

Psychosocial factors also play a central role in dietary decision-making. Managing T1D places a significant emotional burden on parents and caregivers due to persistent uncertainty, continuous monitoring demands, and fear of both short- and long-term complications. In a large multicenter study, Saßmann et al. identified emotional burden as the most substantial and enduring aspect of parental stress, outweighing daily and physical strains and persisting across the child’s developmental stages [43]. In this context, the KD is often viewed not only as a nutritional strategy but also as a means of psychological containment, offering structure and predictability in an otherwise demanding condition.

Some families describe the KD as a way to reclaim autonomy within a healthcare system they experience as rigid or impersonal. The biomedical model of diabetes care, which emphasizes carbohydrate exchange lists, prescribed insulin regimens, and clinic-defined targets, may feel alienating or paternalistic to some caregivers. Adopting a KD can therefore be perceived as an empowering act that aligns management with a child’s everyday lived experience rather than strictly with clinical protocols [37].

However, adherence to the diet may also introduce tension within the family, particularly in households with adolescents. Teenagers may resist dietary restrictions, especially when these limitations interfere with social eating or contribute to food-related anxiety. In addition, highly restrictive diets, if not carefully supervised, may increase the risk of disordered eating behaviors in populations already vulnerable to food-related control dynamics. For parents, maintaining the diet can become an additional source of stress, further straining family relationships [44].

These findings suggest that parental decision-making is influenced not only by clinical outcomes but also by perceived control, emotional burden, and dissatisfaction with conventional management strategies. Importantly, many reported motivations are based on short term glycemic metrics or anecdotal experiences rather than long-term safety data. This highlights a potential disconnect between caregiver priorities and the current evidence base, which may contribute to the adoption of dietary practices that are not fully aligned with established pediatric guidelines.

## 6. Potential Metabolic Effects of the Ketogenic Diet in Pediatric Type 1 Diabetes

### 6.1. Glycemic Outcomes

Recent case series and observational studies in pediatric T1D have reported short-term improvements in glycemic outcomes with ketogenic dietary patterns, including reduced postprandial glucose excursions, greater time within target glycemic range, and lower total daily insulin requirements [5,10,45]. These findings suggest that carbohydrate restriction may reduce glycemic variability and facilitate tighter metabolic control in selected patients. The potential benefits of ketogenic dietary patterns in pediatric T1D are summarized in Table 1. However, such improvements are frequently accompanied by an increased risk of hypoglycemia. Restriction of dietary carbohydrate reduces hepatic glycogen stores and may impair counterregulatory hormonal responses, thereby predisposing children and adolescents to more frequent and occasionally prolonged hypoglycemic episodes [10,46]. CGM data from individuals following very-low-carbohydrate or ketogenic dietary approaches show a greater proportion of time spent in hypoglycemia than those on standard dietary regimens, even when overall HbA1c values remain comparable [10]. This table summarizes the potential benefits of the KD in children and adolescents with T1D.

Taken together, these findings suggest that KDs may offer clinically meaningful improvements in short-term glycemic control, particularly in highly motivated families with access to meticulous monitoring. Nonetheless, the magnitude and sustainability of these potential benefits remain uncertain, largely limited by the predominance of small observational studies and case series, which lack control groups and long-term follow-ups. The inconsistencies in dietary definitions and variability in insulin management strategies may further complicate comparisons across studies and restrict the generalizability of reported glycemic benefits.

A central metabolic consequence of sustained carbohydrate restriction is the depletion of glycogen reserves, which limits the capacity to mount an effective physiological response to hypoglycemia and increases vulnerability during intercurrent illness or periods of increased physical activity [47]. Reduced glycogen availability directly results from chronic limitation of carbohydrate intake and may compromise recovery from hypoglycemia, particularly in pediatric patients who have higher baseline metabolic demands. Case series in children with T1D on restrictive carbohydrate diets have documented endocrine and metabolic disturbances associated with depleted glycogen stores, including impaired hypoglycemia awareness and difficulty in correcting hypoglycemic events [47]. Furthermore, inadequate insulin dosing or illness in the setting of ketogenic dietary patterns may increase the risk of DKA, underscoring the need for careful monitoring and individualized insulin adjustment [5,47]. Clinically, this risk should be interpreted in the context of the distinction between physiological nutritional ketosis and DKA, particularly in terms of ketone levels, glycemia, and acid-base status.

Although short-term glycemic improvements are frequently reported, long-term safety data remain limited. Concerns persist regarding potential adverse effects on linear growth, bone mineralization, lipid profiles, and overall cardiovascular risk in children and adolescents who adhere to ketogenic or very-low-carbohydrate dietary regimens [4,15]. Existing clinical guidance emphasizes that restrictive dietary approaches in pediatric T1D should be undertaken only under close multidisciplinary supervision, including endocrinology, nutrition, and behavioral health support, with ongoing assessment of metabolic control, growth parameters, and psychosocial well-being [4,15]. Further research is needed to clarify the long-term metabolic and developmental consequences of ketogenic dietary patterns in this population and to inform evidence-based clinical recommendations.

### 6.2. Lipid Metabolism and Cardiovascular Risk

A ketogenic dietary pattern in pediatric patients with T1D has been associated with increases in low-density lipoprotein (LDL) cholesterol and total cholesterol, while triglyceride levels may decline. Evidence from meta-analyses and pediatric cohort studies indicates that ketogenic nutritional approaches tend to elevate LDL cholesterol and total cholesterol concentrations while reducing triglycerides, a pattern that is likely related to reduced carbohydrate intake and increased dietary fat consumption [48,49].

Substantial concern exists regarding the quality and composition of dietary fat in this context. The AAP recommends that when carbohydrate intake is reduced, saturated fats should be replaced with polyunsaturated and monounsaturated fats to reduce cardiovascular risk [4]. The ADA similarly advises limiting saturated fat intake to less than 7% of total daily caloric intake and restricting dietary cholesterol to less than 200 milligrams per day in children and adolescents with diabetes, given their classification in the highest cardiovascular risk category [6].

The long-term cardiovascular implications of ketogenic dietary patterns in pediatric T1D remain uncertain. Although some adult case reports suggest relatively favorable cardiovascular profiles in individuals who achieve sustained glycemic control with ketogenic nutrition, pediatric evidence remains limited and raises concern regarding persistent dyslipidemia and potential atherogenic risk [49,50]. Current recommendations support annual fasting lipid profile assessment in children who follow ketogenic dietary patterns, with more frequent monitoring when dyslipidemia is present or emerging [4,15].

## 7. Impact on Growth and Physical Development

Normal growth during childhood is tightly linked to sufficient energy and protein intake, which may be difficult to achieve in dietary regimens that substantially restrict food choices. The marked carbohydrate restriction characteristic of KDs may inadvertently compromise total caloric intake in children with T1D, especially when food acceptance is poor or appetite is blunted, risking growth deceleration and suboptimal weight gain [4]. While protein intake is often maintained or even increased in some ketogenic regimens, the balance between protein adequacy and excessive fat-derived energy remains inconsistent and is largely shaped by parental dietary strategies [51]. Chronic energy deficits, rather than the specific macronutrient composition, may contribute to impaired growth and reduced lean mass accrual. Such concerns are particularly heightened in younger children, who require more energy relative to body size and have reduced physiological reserves to compensate for inadequate nutrition [51,52,53].

The interplay between nutrition, endocrine signaling, and overall metabolic health shapes linear growth trajectories. During periods of rapid growth, prolonged dietary restriction has been associated with impaired height velocity in some pediatric populations [53,54]. Several mechanisms have been proposed linking KDs to altered linear growth, including diminished insulin exposure, perturbations in insulin-like growth factor 1 (IGF-1) signaling, and chronic low-grade energy deficiency [55]. In pediatric T1D populations, the impact of these factors may be further compounded by pre-existing glycemic variability or suboptimal insulin dosing [4,56]. While immediate growth impairment may be subtle, prolonged dietary restriction could result in clinically significant deviations from expected growth trajectories, reinforcing the importance of longitudinal follow-up and careful monitoring [51,54].

Puberty is a metabolically intensive and energy-demanding developmental period that is highly sensitive to nutritional status and metabolic control. Both sufficient energy availability and coordinated endocrine activity are necessary to support normal pubertal onset and progression. Dietary patterns that induce chronic ketosis may modulate the hypothalamic-pituitary-gonadal axis by altering leptin, insulin, and gonadotropin secretion [57,58]. Delayed puberty among children with T1D has been associated with inadequate metabolic control, particularly poor glycemic control, and the added impact of restrictive diets may further disrupt pubertal timing [4,56,59]. Although direct evidence regarding pubertal development in children with T1D treated with KDs is limited, concerns have been raised about delayed menarche, altered pubertal progression, and impaired acquisition of peak bone mass [29,60,61]. These issues merit special attention during adolescence, given heightened nutritional demands and psychosocial influences that may challenge dietary adherence.

Knowledge regarding the impact of KDs on growth can be extrapolated from pediatric epilepsy populations, in whom these diets are frequently administered long-term under medical supervision. Reports from multiple studies in pediatric epilepsy populations have documented reduced height velocity, with variable degrees of catch-up growth following diet discontinuation [52,62]. Importantly, growth impairment observed in epilepsy cohorts has been associated with longer diet duration, greater caloric restriction, and younger age at initiation [52,54,62,63]. However, translating these findings to children with T1D requires caution, as epilepsy-specific ketogenic regimens are generally more stringent and are frequently administered to children whose comorbid neurological conditions may independently affect growth. Collectively, these data suggest the potential impact of KDs on normal physical development, underscoring the need for individualized risk-benefit evaluation when KDs are considered beyond established clinical indications [4]. Much of the available evidence is derived from pediatric epilepsy populations with differing clinical contexts and stricter dietary protocols, and its applicability to children with T1D requires cautious interpretation. Additionally, these studies are frequently observational and subject to confounding factors such as underlying disease severity, comorbidities, and nutritional status, which may bias reported growth outcomes.

## 8. Neurocognitive and Psychosocial Considerations

The neurocognitive and psychosocial implications of ketogenic and low-carbohydrate dietary patterns in pediatric T1D require careful consideration within the context of the developing brain and the complex behavioral demands of diabetes management. Most available data regarding neurocognitive outcomes are derived from pediatric epilepsy cohorts or theoretical considerations rather than direct studies in children with T1D. The absence of controlled prospective studies in pediatric T1D presents uncertainty regarding causality and limits the strength of neurocognitive conclusions.

The AAP notes that the pediatric brain has a high glucose requirement, and sustained carbohydrate restriction may theoretically influence attention, learning, and cognition, although direct evidence of cognitive harm in children with T1D remains limited and largely extrapolated from epilepsy populations and experimental models [4,29,64,65]. Studies in pediatric epilepsy suggest that ketogenic dietary therapy does not produce clear short-term cognitive or behavioral deterioration and may even show modest cognitive improvement, yet confidence in these findings is constrained by high attrition rates and concerns regarding undernutrition and psychosocial adjustment [4,66]. In children with T1D, both hypoglycemia and hyperglycemia are independently associated with structural brain changes and deficits in executive function, memory, and attention, particularly during periods of rapid neurodevelopment, and the potential for increased hypoglycemia with ketogenic dietary adherence may further heighten neurocognitive vulnerability [10,64,67].

Beyond neurocognitive considerations, restrictive dietary regimens can alter the relationship with food and are associated with elevated risk of disordered eating behaviors in youth with T1D, including deliberate insulin omission and maladaptive preoccupation with dietary control, both of which adversely affect metabolic and psychological outcomes [4,5,68]. Qualitative and observational data further indicate that strict dietary rules may contribute to diabetes related distress, depressive and anxiety symptoms, and diminished quality of life, particularly when adherence interferes with social eating, peer interaction, and participation in routine activities [66,68]. Increased parental stress and the potential for social isolation within families following rigid dietary regimens have also been reported, underscoring the importance of routine screening for disordered eating and mental health concerns, along with comprehensive multidisciplinary monitoring and individualized risk assessment when considering ketogenic dietary approaches in pediatric T1D [4,66,68].

## 9. Micronutrient Deficiencies and Gastrointestinal Effects

The KD is structured around strict carbohydrate limitations, often excluding fruits, whole grains, legumes, and various vegetables. Although effective in inducing nutritional ketosis, this dietary pattern narrows food diversity and may predispose individuals to micronutrient insufficiency. For children and adolescents with T1D, where dietary management and adequate nutrition underpin both metabolic stability and physical development, the implications for individual micronutrient status necessitate careful consideration.

### 9.1. Risk of Micronutrient Deficiencies

Children and adolescents with T1D who adhere to KDs may be at risk of deficiencies in fiber, calcium, vitamin D, iron, magnesium, zinc, and selenium [15,29,68]. Reduced consumption of legumes, whole grains, and fruits substantially lowers dietary fiber intake, which negatively affects glycemic stability, satiety regulation, and cardiometabolic health in T1D. Studies have shown that high-fiber diets are related to improved HbA1c, fewer hypoglycemic episodes, and reduced glycemic variability [69,70]. Excluding fiber-rich foods in KDs risks GI dysfunction and suboptimal metabolic outcomes. However, the evidence base derived from dietary modeling studies, observational reports, and extrapolation from non-T1D populations constrains the ability to reliably quantify the prevalence and clinical impact of micronutrient deficiencies in pediatric T1D.

When dairy products are limited or excluded in restrictive KD regimens, calcium and vitamin D intake may be insufficient. During critical growth phases, inadequate consumption of these nutrients may impair bone mineralization and adversely affect skeletal development, hindering bone mass accrual. This concern is heightened in T1D, as evidence indicates significantly reduced bone mineral density and bone mineral content relative to healthy peers [71,72,73,74]. Another potential concern is iron deficiency when reductions in iron-fortified grains and plant-based sources are not adequately compensated from bioavailable or animal-based heme iron. Subclinical iron deficiency may negatively influence cognitive performance, physical endurance, and immune function, thereby affecting optimal pediatric development [75]. By restricting plant-based sources, magnesium intake may become suboptimal. Given magnesium’s integral role in insulin-mediated glucose regulation, neuromuscular function, and musculoskeletal health, insufficient intake may further destabilize metabolic homeostasis and impaired bone health in children with T1D [71].

Zinc deficiency may stem from the substantial restriction or even exclusion of carbohydrate-based, zinc-rich foods such as whole grains, legumes, and certain fruits, compounded by additional protein restrictions that limit meat and seafood intake [4]. Moreover, some ketogenic formulations incorporate plant-derived fats or protein sources rich in phytates, which can impair zinc absorption, while prolonged ketosis may exacerbate the effect by compromising gut function and reducing zinc bioavailability [76,77,78,79]. Given its fundamental role in deoxyribonucleic acid (DNA) synthesis, cell proliferation, oxidative stress, immune responses, and hormonal regulation, including IGF-1 and growth hormone, zinc deficiency can adversely affect linear growth and pubertal maturation, even when the deficiency is mild. Clinical manifestations may include growth retardation, delayed puberty, immune dysfunction with frequent infections, poor wound healing, hair loss, alopecia, hypogeusia, and perioral or acral dermatitis, and dermatologic abnormalities, collectively undermining overall well-being and development [79,80,81].

KDs commonly eliminate grains and may restrict intake of nuts and seafood, which are key sources of selenium [4,82]. The high-fat nature of KDs may disrupt selenium transport and selenoprotein biosynthesis, whereas routine selenium supplementation is uncommon unless deficiencies are actively monitored and detected [4,83,84,85]. Selenium deficiency compromises antioxidant capacity, heightens oxidative stress, and interferes with thyroid hormone metabolism, particularly the selenoprotein-mediated conversion of thyroxine (T4) to triiodothyronine (T3), resulting in diminished metabolic efficiency and indirect effects on growth. Selenium deficiency may manifest as myopathy, fatigue, lethargy, increased oxidative stress, and in rare instances, cardiomyopathy [82,86,87,88].

### 9.2. Constipation and Gut Microbiota Alterations

Constipation is among the most commonly reported GI adverse events of KD in pediatric populations, generally due to reduced dietary fiber intake, altered gut motility, and lowered stool bulk [4]. As glycemic variability in T1D can contribute to GI dysmotility, further restrictive dietary patterns may aggravate these symptoms [4]. In addition to functional constipation, KDs may alter the gut microbiota. Carbohydrate restriction reduces fermentable fiber substrates for commensal bacteria, thereby decreasing short-chain fatty acid production and altering microbial diversity [89,90,91]. Despite hypotheses linking alterations in the microbiome to KDs’ therapeutic benefits in epilepsy, the long-term relevance and impact of such changes in otherwise healthy children with T1D have not been established [92]. With growing evidence recognizing the interplay between gut microbial composition, immune regulation, and metabolic homeostasis, chronic dysbiosis may carry unintended consequences for pediatric development and metabolic health [91,92,93].

### 9.3. Need for Supplementation and Monitoring

Given these recognized risks, micronutrient supplementation is recommended in pediatric KD regimens, as established in refractory epilepsy care. In the absence of formal clinical indications, a similar degree of vigilance and scrutiny is required for children with T1D adopting KD. Baseline and ongoing assessment of anthropometric measures, dietary intake, nutritional status, and laboratory indicators, including iron, vitamin D, and, if indicated, bone health indices, should be incorporated into standard follow-up [94]. Furthermore, close dietetic supervision and guidance are crucial for ensuring sufficient protein intake for growth, adequate consumption of non-starchy vegetables for fiber and micronutrients, and the individualization of supplementation strategies tailored to age, sex, and dietary adequacy to mitigate potential adverse effects [95]. Without careful oversight from a multidisciplinary team, persistent micronutrient deficiencies and GI complications in children and adolescents with T1D may compromise metabolic function and long-term developmental outcomes.

## 10. Clinical Challenges and Ethical Considerations

By integrating metabolic, developmental, psychosocial, and ethical aspects, this review provides a multidimensional assessment that extends beyond traditional outcome-focused analyses. The clinical implementation of ketogenic dietary patterns in pediatric T1D presents substantial challenges related to limited evidence, monitoring complexity, and ethical considerations in shared decision-making. A major barrier is the lack of high-quality pediatric-specific data, which restricts confidence in both safety and efficacy. Much of the current evidence is extrapolated from adult cohorts or pediatric epilepsy populations, and available pediatric studies are typically small, heterogeneous, and short-term, making it difficult to draw robust conclusions about long-term outcomes in children with T1D [4,5,10,15,29]. This uncertainty is compounded by theoretical and observed risks, including growth deceleration, impaired bone health, hypoglycemia, dyslipidemia, and disordered eating behaviors, all of which require careful longitudinal assessment and multidisciplinary oversight [5,10,15,29]. The AAP recommends intensive monitoring that includes frequent laboratory evaluation, growth tracking, and routine mental health screening, yet acknowledges that the resource demands associated with continuous glucose monitoring, specialized laboratory tests, and comprehensive dietary supervision may strain routine clinical practice and complicate safe implementation [4].

Practical challenges in routine care are further amplified by the absence of standardized clinical protocols for KD initiation and follow-up in pediatric T1D. Clinicians must simultaneously monitor glycemic variability, ketone trends, growth parameters, bone health, cardiometabolic risk markers, and overall nutritional adequacy, often requiring more frequent and specialized assessments than standard diabetes care [15,96]. Variability in clinical practice and the lack of consensus on ketone targets or management strategies create additional uncertainty, particularly as emerging diabetes technologies alter monitoring approaches and expectations [15,96].

Ethical considerations center on balancing parental autonomy with the obligation to protect child welfare and long-term development. Families may pursue ketogenic dietary interventions based on perceived metabolic benefits, social influences, or dissatisfaction with conventional management strategies, yet clinicians must ensure that dietary modifications do not compromise growth, psychosocial well-being, or overall health [15,97]. The AAP emphasizes the importance of open dialog, shared decision-making, and regular follow-up with structured nutritional oversight to mitigate potential risks while respecting family preferences [4].

Effective clinician and family communication is therefore central to safe care. Survey data indicate that some families adopting KDs perceive judgment or mistrust from diabetes care teams, which can undermine therapeutic alliance and adherence to monitoring recommendations [4]. Professional societies highlight the importance of meeting families where they are, maintaining nonjudgmental communication, and providing individualized education and decision support to facilitate collaborative management and optimize both clinical and psychosocial outcomes for pediatric patients with T1D [15,97].

Ethical considerations in this context require careful balancing of parental autonomy with the obligation to protect the child’s best interests. While caregivers play a central role in dietary decision-making, children with T1D represent a vulnerable population in whom restrictive dietary practices may carry risks to growth, metabolic stability, and psychosocial well-being [4,5,15,29]. Clinicians must therefore navigate situations in which parental preferences may diverge from evidence-based recommendations, particularly given the limited and heterogeneous nature of current data [5,10,15]. In this setting, professional guidance emphasizes the importance of safeguarding long term developmental outcomes while maintaining a collaborative and nonjudgmental approach to care [4,15]. Potential risks such as disordered eating behaviors, psychosocial stress, and impaired quality of life further reinforce the need for careful ethical consideration and ongoing monitoring [4,66,68]. These challenges highlight the importance of shared decision-making, transparent communication of risks and uncertainties, and prioritization of the child’s overall health and development.

In addition, pediatric ethical frameworks emphasize that parental decision-making authority is limited by the requirement that choices support the child’s health and long-term developmental interests. When dietary interventions introduce risks to growth, metabolic stability, or psychological well-being, clinicians have a duty to advocate for the child’s welfare, even if this involves respectfully challenging parental preferences.

The child’s emerging autonomy also plays a role. Older children and adolescents may have their own views regarding restrictive diets, and incorporating their perspectives into shared decision-making is considered best practice in pediatric care.

Overall, while parental involvement is essential, the child’s best interest remains the guiding principle. Clinicians must balance respect for family values with proactive safeguarding of growth, psychosocial health, and long-term developmental outcomes, particularly when evidence is limited and potential risks are significant.

## 11. Practical Considerations for Clinicians

The AAP recommends a multidisciplinary, closely supervised approach when counseling families considering a ketogenic dietary pattern for a child with T1D, with regular follow-up by a pediatric endocrinologist, diabetes educator, and registered dietitian. Counseling should include a clear discussion of potential risks, such as nutritional deficiencies, impaired growth, dyslipidemia, and an increased risk of hypoglycemia and DKA, as well as the importance of maintaining adequate caloric intake and a balanced macronutrient composition, with emphasis on unsaturated fat sources rather than saturated fats [4]. The major negative effects on metabolic, developmental, and psychosocial aspects in a child with T1D following the ketogenic dietary pattern are summarized in Table 2. Families should be informed that although short-term improvements in glycemic variability and time in range may occur, pediatric evidence remains limited and largely derived from small or heterogeneous studies, and concerns persist regarding possible adverse effects on growth, bone health, and long-term metabolic outcomes [5,13,14,15]. Table 2 presents the potential negative effects of the ketogenic diet in the childhood population with T1D.

Clinicians should identify and clearly communicate warning signs that require prompt reassessment or intervention. These include:

- Growth delay or impaired linear growth.
- Delayed pubertal development.
- Significant or unexplained weight change.
- Symptoms of micronutrient deficiency (for example fatigue, anemia, dermatologic changes).
- Recurrent or severe hypoglycemia.
- Dyslipidemia, particularly rising low density lipoprotein cholesterol.
- Evidence of disordered eating behaviors, including insulin omission or excessive preoccupation with dietary restriction [4,5,42].

In addition, acute illness or missed insulin doses warrant urgent evaluation, as the risk of diabetic ketoacidosis is increased and nutritional ketosis may obscure early recognition of metabolic decompensation [4,98].

When a child with T1D is already following a ketogenic or very-low-carbohydrate dietary pattern, clinicians should adopt a nonjudgmental and collaborative approach to maintain therapeutic alliance and ensure patient safety [4,15]. Initial evaluation should include detailed assessment of dietary composition, caloric adequacy, insulin regimen, and glycemic patterns, including time in range and frequency of hypoglycemia [4,14,42]. Growth parameters, including weight, height, and growth velocity, should be carefully monitored, as restrictive dietary patterns have been associated with impaired linear growth and altered endocrine signaling in pediatric populations [4,51,52,53,54,55]. In addition, clinicians should assess for micronutrient deficiencies, given the documented risk of inadequate intake of vitamins and minerals in ketogenic dietary patterns [15,29,68].

Where continuation of the diet is considered, close multidisciplinary monitoring is essential, with regular reassessment of metabolic control, lipid profile, nutritional status, and psychosocial well-being [4,15].

Clinicians should be vigilant for warning signs that may indicate adverse effects or the need to modify or discontinue the diet. Key red flags include:

- Impaired linear growth or reduced height velocity, reflecting potential energy imbalance and altered growth factor signaling [51,52,53,54,55].
- Delayed pubertal development, which may be influenced by nutritional and endocrine disruption [57,58,59,60].
- Recurrent or severe hypoglycemia, associated with reduced glycogen stores and impaired counterregulatory responses during carbohydrate restriction [10,46,47].
- Dyslipidemia, particularly elevated low density lipoprotein cholesterol, which has been reported in association with ketogenic dietary patterns [48,49].
- Evidence of micronutrient deficiency, including iron, vitamin D, magnesium, zinc, or selenium deficiency due to dietary restriction [15,29,68].
- Symptoms of disordered eating or excessive dietary restriction, which are of particular concern in pediatric T1D populations [4,5,68].
- Recurrent ketosis or clinical concern for diabetic ketoacidosis, particularly during illness or inadequate insulin dosing [5,47].

The presence of these findings should prompt reassessment of dietary safety and consideration of dietary modification or discontinuation.

A ketogenic or very low carbohydrate dietary pattern should generally be discouraged in the following situations:

- Inadequate growth or existing growth failure [4,51,52,53,54].
- Poor adherence to insulin therapy or unreliable glucose and ketone monitoring [4,96].
- History of recurrent diabetic ketoacidosis or increased risk of metabolic decompensation [5,47].
- Presence of disordered eating behaviors or significant psychosocial risk [4,5,68].
- Limited access to multidisciplinary follow up, including dietetic and endocrinology support [4].
- Inability to ensure adequate caloric intake or nutritional completeness [4,15].

In these contexts, restrictive dietary patterns may pose greater risk than benefit and should be avoided.

For many children and adolescents with T1D, less restrictive dietary approaches, including moderate carbohydrate reduction, may provide improvements in glycemic variability while minimizing risks to growth, nutritional status, and psychosocial well-being [4,14]. Individualized nutrition therapy aligned with established guidelines remains the preferred approach in pediatric diabetes care [11,12].

## 12. Gaps in Evidence and Future Research Directions

The evidence base informing the use of ketogenic and very low carbohydrate diets in pediatric T1D remains limited and heterogeneous. Much of the available data is derived from small observational studies, adult populations, or pediatric epilepsy cohorts, where ketogenic therapies are used for different clinical indications and under structured medical supervision. As a result, extrapolation of these findings to children with T1D should be interpreted with caution. While physiological mechanisms may suggest potential metabolic and developmental effects, direct evidence in pediatric T1D populations is sparse, and the long-term safety and clinical implications remain incompletely understood.

### 12.1. Lack of Long-Term Prospective Studies

Despite growing parental interest and emerging short-term findings of glycemic improvement, the evidence base for ketogenic and VLCDs in pediatric T1D remains limited in scope, duration, and methodological quality [97]. The preceding sections delineate concerns spanning growth, skeletal health, lipid metabolism, micronutrient status, hypoglycemia vulnerability, psychosocial burden, and long-term cardiometabolic health. However, to date, much of the existing knowledge derives from small, heterogeneous observational studies and indirect extrapolation from pediatric epilepsy or adult T1D cohorts [9,13,15,69,97]. The scarcity of robust pediatric prospective data limits precise quantification of long-term risk-benefit profiles and precludes determining whether short-term glycemic improvements translate into sustainable, developmentally safe outcomes.

### 12.2. Methodological Gaps and Need for Standardized Definitions

A major methodological gap lies in the inconsistent and poorly defined terminology for dietary intake. Terms such as “ketogenic,” “very-low-carbohydrate,” and “low-carbohydrate” in pediatric T1D studies are often applied interchangeably and without clear specification of macronutrient thresholds or ketosis metrics [9,13,98]. This concern is supported by recent methodological critiques, which highlight substantial heterogeneity in dietary definitions, study design, and outcome interpretation, ultimately challenging the validity and comparability of pooled analyses and findings across KD research [99,100]. Future research should use uniform, standardized, age-specific definitions of carbohydrate intake, expressed both in grams per day and as a percentage of total energy. Detailed reporting of dietary fat composition, protein adequacy, micronutrient supplementation practices, and systematic measurement of ketosis biomarkers is necessary to enhance reproducibility, interpretability, and comparability of research findings [13,15,97,98]. Without standardized definitions and reporting frameworks, the synthesis of outcomes and the development of clinical guidance for pediatric populations will remain limited.

As supported by the AAP, there is a need for long-term, prospective pediatric cohort studies in children or adolescents with T1D who adhere to ketogenic or low-carbohydrate diets, specifically evaluating predefined endpoints for metabolic, growth, and safety [4]. This would allow longitudinal assessment of growth velocity, anthropometric measurements, lipid profiles, bone mineral density, and glycemic variability over time, while adjusting for confounders such as baseline metabolic control, insulin therapy, socioeconomic conditions, and pubertal stage [4]. Although randomized controlled trials may not be feasible for ethical and practical reasons, well-structured multicenter prospective observational studies with matched comparison groups could significantly strengthen the evidence base for clinical guidance and developmental safety in pediatric patients on ketogenic or low-carbohydrate diets [10,15,29,101].

### 12.3. Developmental, Endocrine, and Neurocognitive Outcomes

The long-term effects of carbohydrate restriction on growth, endocrine function, and neurocognitive development in children with T1D remain poorly understood. However, the AAP recommends systematic measurement of height velocity, Tanner staging, and insulin-like growth factor-related markers, along with dual-energy X-ray absorptiometry (DXA) assessments for children following restrictive diets for more than two years [4]. Monitoring these developmental parameters is crucial, as minor deviations in growth, pubertal timing, or bone accrual may only become evident over the long term, and early intervention is imperative.

For children and adolescents with T1D on ketogenic or low-carbohydrate diets, the ADA recommends structured investigation of neurocognitive outcomes [6,102]. Validated test batteries (e.g., MOXO-continuous performance test, Wechsler Intelligence Scale for Children—Revised) can be employed to evaluate attention, memory, executive function, and processing speed, integrated with CGM data (e.g., time in range, hypoglycemia exposure) to explore relationships between metabolic patterns, such as hypoglycemia or prolonged ketosis, and cognitive trajectories [103,104,105]. Additionally, incorporating academic performance and quality-of-life indicators would contextualize functional outcomes relevant to everyday life and long-term development with regard to dietary interventions [4,102].

### 12.4. Psychosocial and Behavioral Outcomes

The psychosocial impact of chronic carbohydrate restriction in pediatric T1D has been documented by studies indicating increased risk for diabetes-related stress, anxiety, depression, and disordered eating behaviors, particularly among adolescents. The ADA recommends routine screening using validated tools such as the Revised Children’s Anxiety and Depression Scale (RCADS) and Eating Attitudes Test (EAT-40) for depression and anxiety, and the Diabetes Eating Problem Survey-Revised (DEPS-R) for disordered eating [6,102]. By combining quantitative metabolic measures (e.g., HbA1c, CGM data) with qualitative interviews of children, adolescents, and caregivers in a mixed-methods approach, researchers may better understand adherence patterns, caregiver motivations, family influences, and unintended psychosocial consequences [106,107]. Clarifying these behavioral factors is important for situating metabolic results within the framework of lived experiences and for informing supportive, evidence-based counseling.

### 12.5. Sociocultural and Digital Influences

Digital media and online peer networks play a substantial role in shaping parental perceptions and decision-making regarding KD content in pediatric T1D. Although parental trust in online resources varies depending on perceived suitability and credibility, the collective effect of social media groups and forums may outweigh the child’s input in certain aspects, influencing parents’ trust in medical advice, perceived safety, efficacy of dietary interventions, and implementation patterns [108]. Peer–parent interactions and online communities can affect daily treatment decisions by serving as primary sources of non-medical information and emotional support. When reinforced by peer endorsement and perceived success stories, these influences may foster skepticism toward clinical guidance and create discordance with professional advice [109]. Interdisciplinary collaboration across pediatric endocrinology, diabetology, dietetics, and psychology could help counter misinformation, guide evidence-based dietary decisions, and reinforce clinician-family trust amid an evolving sociocultural and digital landscape. The AAP recommends maintaining open dialog, regular medical follow-up, and multidisciplinary surveillance to address both physiological and psychosocial risks associated with ketogenic or carbohydrate-restricted diets [4,110].

### 12.6. International Collaboration and Registry Development

Given the small cohorts of sustained ketogenic dietary use in pediatric T1D within individual centers, collaborative efforts across multiple centers and countries are required to produce robust, statistically meaningful datasets. Developing a standardized global registry for pediatric T1D patients following ketogenic or carbohydrate-restricted diets would permit systematic, longitudinal surveillance of growth, metabolic parameters, pubertal development, bone health, micronutrient status, neurocognition, and psychosocial functioning [4]. Such coordinated infrastructure would improve the detection of adverse events and facilitate hypothesis-driven investigations and subsequent controlled studies. Progress in this field calls for methodological standardization, long-term developmental follow-up, incorporation of psychosocial and neurocognitive endpoints, and coordinated international research efforts. In the absence of rigorous prospective data, clinical guidance and treatment decisions will remain based on precautionary extrapolation rather than definitive pediatric outcomes [6,101,111,112].

## 13. Conclusions

In pediatric T1D, ketogenic and VLCDs may improve short-term glycemic stability and lower insulin doses, outcomes consistent with parental motivations. Nevertheless, these perceived metabolic benefits must be weighed against safety concerns, including hypoglycemia, dyslipidemia, micronutrient deficiencies, growth disorder or retardation, pubertal delay, skeletal integrity, and psychosocial stress. During periods of rapid growth and neuroendocrine maturation, significant carbohydrate restriction warrants prudence. Nutritional management for children with T1D should address not only glycemic control but also support healthy physical, cognitive, and psychosocial development. When restrictive carbohydrate diets are adopted, individualized, evidence-based nutrition counseling and close oversight by a multidisciplinary team are necessary to ensure safety. Shared decision-making should align with established pediatric guidelines and recommendations, and prioritize long-term developmental considerations. More rigorous, long-term prospective pediatric studies are needed before broader clinical applications regarding ketogenic dietary approaches can be widely recommended in children with T1D. This review offers a clinically oriented synthesis integrating caregiver behavior, real-world dietary practices, and developmental considerations, providing a framework for both clinical counseling and future research.

## Figures and Tables

**Table 1:** Overview of the potential benefits of a ketogenic diet in children and adolescents with T1D.

Potential Benefits	Description	References
	**Objective clinical outcomes**	
Reduced postprandial glucose excursions	Carbohydrate restriction reduces postprandial glucose spikes, resulting in more stable postprandial glycemia.	[5,35]
Improved glycemic stability	Some observational studies and case series describe reduced glycemic variability and greater time in range in individuals following KDs.	[4,5,10,35]
Lower HbA1c levels	Some studies report lower HbA1c levels in individuals with T1D following VLCDs.	[35]
Reduced insulin requirements	Lower carbohydrate intake decreases the need for bolus insulin and total daily insulin doses.	[5,37]
Potential reduction in insulin-associated weight gain	Lower insulin doses may reduce the risk of insulin-related weight gain.	[38]
	**Perceived or caregiver-reported benefits**	
Simplified insulin management	Restricting carbohydrate-containing foods may simplify carbohydrate counting and insulin adjustments.	[37]
Reduced frequency of hyperglycemia corrections	Caregivers and families report fewer correction boluses due to more predictable glycemic responses.	[4,35]
Greater perceived sense of control over diabetes management	Some caregivers and families report improved predictability of glucose patterns and reduced alarm burden on CGM devices.	[4]

KD—ketogenic diet; HbA1c—glycated hemoglobin; T1D—type 1 diabetes; VLCD—very-low-carbohydrate diet; CGM—continuous glucose monitoring.

**Table 2:** Summary of the potential negative effects of the ketogenic diet in pediatric T1D.

Potential Risk/Adverse Effect	Description	References
	**Objective clinical risks**	
Hypoglycemia	Reduced glycogen storage and altered regulatory responses may increase susceptibility to hypoglycemia.	[10,46,47]
Diabetic ketoacidosis (DKA)	Illness or inadequate insulin dosing during ketogenic dieting may increase DKA risk.	[5,47]
Dyslipidemia	KDs may increase LDL cholesterol and total cholesterol, raising concerns about cardiovascular risk.	[48,49]
Linear growth impairment	Chronic energy restriction and altered hormonal signaling may impair height velocity in growing children.	[51,52,53,54,55]
Delayed puberty and endocrine effects	Altered insulin, leptin, and gonadotropin signaling may affect pubertal development.	[57,58,59,60]
Impaired bone health	Inadequate calcium and vitamin D intake may impair bone mineralization and peak bone mass accrual.	[71,72,73,74]
Micronutrient deficiencies	Deficiencies in fiber, calcium, vitamin D, iron, magnesium, zinc, and selenium may occur due to dietary restrictions.	[15,29,68]
Gastrointestinal symptoms	Reduced fiber intake and altered gut motility may cause constipation and gastrointestinal discomfort.	[4]
Gut microbiota alterations	Carbohydrate restriction may reduce fermentable fiber and alter gut microbial diversity.	[90,91,92,93]
Neurocognitive vulnerability	Increased hypoglycemia risk and reduced glucose availability may potentially affect attention, memory, and cognition.	[10,64,67]
	**Psychosocial and behavioral risks**	
Disordered eating behaviors	Strict dietary rules may contribute to food-related anxiety, insulin omission, or eating disorders.	[4,5,68]
Psychosocial stress and reduced quality of life	Restrictive diets may interfere with eating behaviors, peer relationships, and family dynamics.	[4,66,68]
Increased parental/caregiver stress and monitoring burden	Implementing strict diets may increase family stress and require intensive monitoring.	[4,66,68]

DKA—diabetic ketoacidosis; KD—ketogenic diet; LDL—low-density lipoprotein.

## Data Availability

No new data were created or analyzed in this study. Data sharing is not applicable to this article.

## Abbreviations

The following abbreviations are used in this manuscript:

KD	Ketogenic diet
T1D	Type 1 diabetes
CGM	Continuous glucose monitoring
ADA	American Diabetes Association
ISPAD	International Society for Pediatric and Adolescent Diabetes
AAP	American Academy of Pediatrics
MNT	Medical nutrition therapy
KDT	Ketogenic diet therapy
CKD	Classic ketogenic diet
MCTKD	Medium-chain triglyceride ketogenic diet
MAD	Modified Atkins diet
LGIT	Low glycemic index treatment
GI	Gastrointestinal
GLUT-1	Glucose transporter protein 1
ASD	Autism spectrum disorder
VLCD	Very-low-carbohydrate diet
HbA1c	Hemoglobin A1c
DKA	Diabetic ketoacidosis
LDL	Low-density lipoprotein
IGF-1	Insulin-like growth factor 1
DNA	Deoxyribonucleic acid
T4	Thyroxine
T3	Triiodothyronine
DXA	Dual-energy X-ray absorptiometry
RCADS	Revised Children’s Anxiety and Depression Scale
EAT-40	Eating Attitudes Test
DEPS-R	Diabetes Eating Problem Survey-Revised
